# The role of structure in regulatory RNA elements

**DOI:** 10.1042/BSR20240139

**Published:** 2024-10-23

**Authors:** Jan-Niklas Tants, Andreas Schlundt

**Affiliations:** 1 Institute for Molecular Biosciences and Biomolecular Resonance Center (BMRZ), Goethe University Frankfurt, Max-von-Laue-Str. 7-9, 60438 Frankfurt, Germany; 2 University of Greifswald, Institute of Biochemistry, Felix-Hausdorff-Str. 4, 17489 Greifswald, Germany

**Keywords:** cis-regulatory elements, dynamics, RNA conformers, RNA structure, RNA-binding proteins, stem-loop

## Abstract

Regulatory RNA elements fulfill functions such as translational regulation, control of transcript levels, and regulation of viral genome replication. *Trans*-acting factors (i.e., RNA-binding proteins) bind the so-called *cis* elements and confer functionality to the complex. The specificity during protein-RNA complex (RNP) formation often exploits the structural plasticity of RNA. Functional integrity of *cis*-*trans* pairs depends on the availability of properly folded RNA elements, and RNA conformational transitions can cause diseases. Knowledge of RNA structure and the conformational space is needed for understanding complex formation and deducing functional effects. However, structure determination of RNAs under *in vivo* conditions remains challenging. This review provides an overview of structured eukaryotic and viral RNA *cis* elements and discusses the effect of RNA structural equilibria on RNP formation. We showcase implications of RNA structural changes for diseases, outline strategies for RNA structure-based drug targeting, and summarize the methodological toolbox for deciphering RNA structures.

## Introduction

RNA, originally believed to only be a transmitter of genetic information, is now recognized as a versatile biomolecule with functions in the regulation of gene expression, viral and bacterial defense mechanisms, and guiding and scaffolding. RNA can adopt complex structures and shows considerable variations in its degree of structure owing to active unfolding *in vivo* [1,2]. Furthermore, a single RNA molecule can occur in sequence isoforms or be dynamic in structure and the level of modifications [3–5]. This heterogeneity and plasticity allow rapid adaptation to varying cellular contexts [6,7]. Although RNAs can be functional on their own, for example, in mediating phase separation based on RNA–RNA interactions [8,9] they are often heavily decorated by proteins. A fine-tuned interaction network with *trans*-acting partners (protein, DNA, or RNA) ultimately governs the function and fate of an RNA. The binding partners exert their RNA-processing function by recognizing RNA *cis*-regulatory elements (*cis* elements) in a sequence- or shape-specific manner, with RNA structure often being the driving force of complex formation [10]. Proteins use a modular architecture of multiple RNA-binding domains (RBD) or (hetero)dimerization to engage with target RNA elements [11–13], thereby enhancing specificity and affinity through an increased interaction network.

The first discovered *cis* elements were short linear motifs [14,15] responsible for translation initiation and mRNA decay. Knowledge of *cis* elements has expanded over the past two decades and elements of diverse size, structure, and complexity are known [16,17]. Some of these *cis* elements are promiscuous regarding their *trans* partners, whereas others have co-evolved to form highly specific *cis*-*trans* pairs [18–20]. Subtle changes in the sequence and, consequently, the structure of highly conserved RNA residues were found to be a key determinant for the onset of diseases such as cancer, neuropathological disorders, or infections [21,22]. Often, RNA structures *per se* are not pathologic, but their equilibria with multiple conformations are, e.g., when equilibria are distorted in mutant forms carrying devastating single nucleotide polymorphisms (SNPs) [23,24]. As a result, binding sites can be obscured or exposed, altering the *trans* interaction network [25]. Therefore, the structural availability of RNA *cis* elements is crucial for the observed function(s). However, the intrinsic heterogeneity and transient interactions of RNA mean capturing the complete conformational landscape of an RNA molecule *in vivo* is challenging and consequently affects the determination of the *cis*-*trans* network and its function.

In this review, we discuss structures and functions of regulatory RNA *cis* elements, focusing on eukaryotic and viral systems; the role of RNA structures in bacteria has been extensively summarized in various articles [7,26,27]. We describe how RNA structure regulates a sequence-specific readout by proteins through linear and folded *cis* elements. We also present common structural features and highlight the crucial role of RNA structure for RNP formation through examples of transient and heterogeneous RNA structures. In addition, we summarize how RNA structural transitions are used to integrate external stimuli. Higher-order structures, for example, internal ribosome entry sites (IRES) often serve as an anchor for extended protein–RNA networks, and the contribution of RNA structures to IRES function is exemplified herein. We showcase disease-associated RNA structures and cover recent advances in RNA-targeting drug design. Finally, we compare methods for determining heterogeneous and dynamic RNA structures *in vitro* and *in vivo*.

## Types of regulatory RNA elements

**Figure 1:**
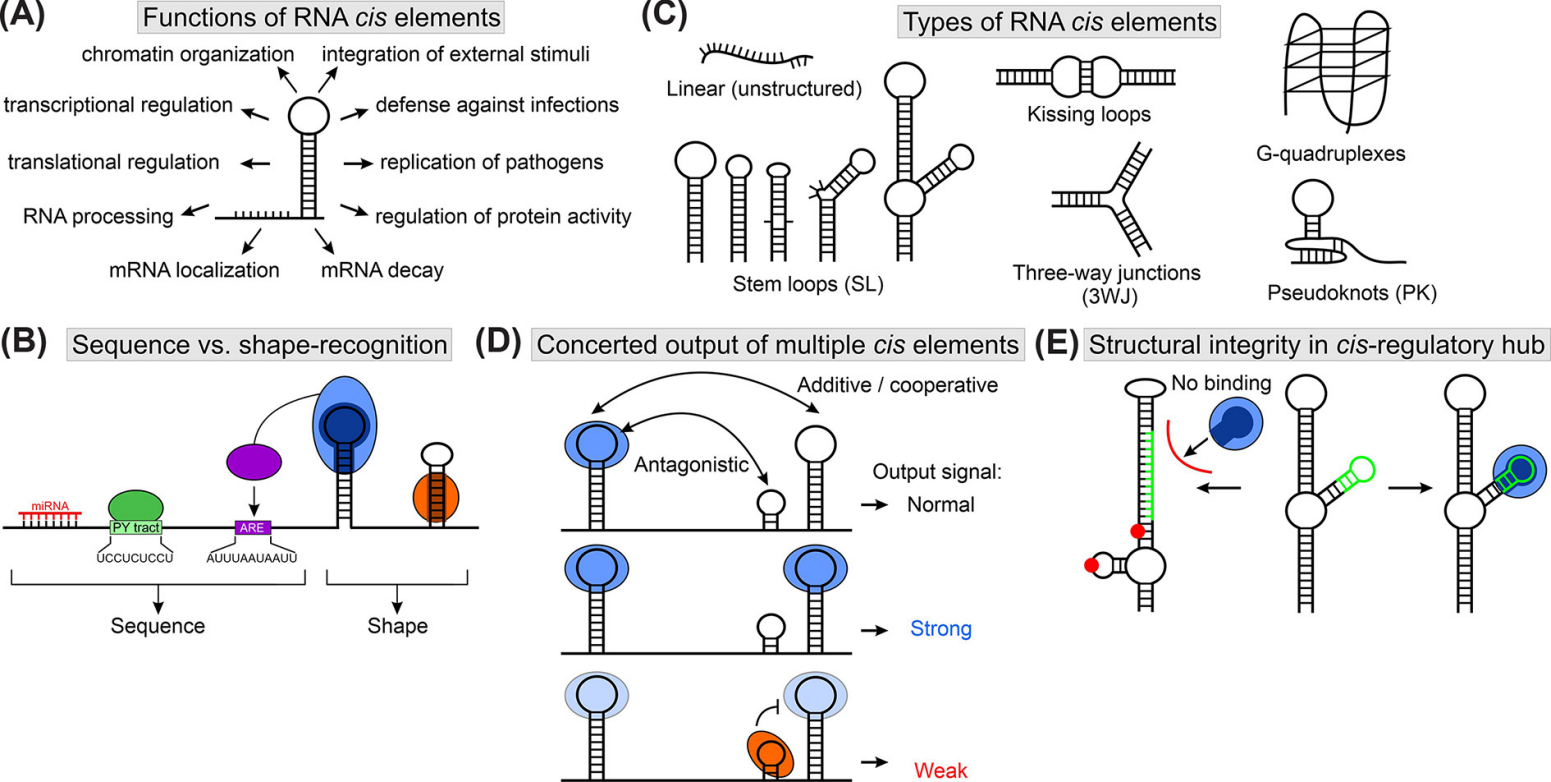
Types and function of RNA *cis*-regulatory elements (**A**) Functions of RNA *cis* elements. (**B**) Linear and structured RNA elements in a cartoon-model RNA. Sequence-specific recognition of RNA target sites can be mediated by proteins, e.g., to poly-pyrimidine (PY) tracts or AU-rich elements (AREs, green and purple), and miRNAs (red). Specialized protein domains can recognize shapes, e.g., stem-loops (blue) or double-stranded regions (orange), through adapted binding pockets (dark blue and orange). The modular architecture of proteins allows integration of multiple specificities, as exemplified by a protein composed of the purple and blue domains, thereby combining sequence and structure specificity. (**C**) Examples of *cis* element structures. Stem-loops (SL) can vary in their stem length and loop size and contain mismatches or bulges that lead to distinct geometries. Complex structures such as branched SLs can be constructed from SLs and bulges. Interaction of two hairpins via their loop regions leads to formation of kissing-loops, often exploited in RNA dimerization. Higher-order structures comprise three-way junctions (3WJ), G-quadruplexes, and pseudoknots (PK). (**D**) *Cis*-regulatory cassette composed of multiple *cis* elements exhibiting additive/cooperative or antagonistic effects for a concerted output. Two hairpin structures can be bound by one or two proteins (blue), leading to a normal or strong output signal, respectively. Binding of a second, antagonistic protein (orange) to a different SL structure counteracts the positive regulatory effect of the other two SLs, leading to a weak output. (**E**) Complex structure of a regulatory hub for exposure of *trans* factor binding site. In a properly folded RNA, the green protein-binding site forms a SL that is bound by the blue protein in a shape-specific manner. Point mutations (shown as red spheres) or conformational equilibria can favor the formation of a second conformation, which traps the protein-binding site in a stable stem structure. Through prevention of hairpin formation, the protein is no longer able to engage with the target RNA.

Regulatory RNA elements are found in the untranslated regions (UTRs) and coding sequences of mRNAs, and in lncRNAs, miRNAs, and viral genomic or subgenomic RNAs. Elements can work in *cis* or in *trans* for potential downstream effects of the RNA in which they are contained, with most regulatory units specifically engaging with *trans* factors. RNA elements have been found by coincidence or through cell-based screening approaches or computational analysis of genomics data and prediction [28–30]. In this section of the review, we summarize the most important and well-studied *cis-*regulatory elements based on their degree of structure (Figure 1 and Table 1).

**Table 1:** Regulatory RNA elements and their structure and function(s). Note that examples are given for interaction partners, functions, and associated diseases in eukaryotes

RNA element	Species	Structure (functional)	Interaction partner	Function	Associated disease	References
**Start codon (AUG or unusual ones)**	All kingdoms of life	Linear	Ribosome, initiation complex	Encodes start methionine during protein translation	Alternate protein production	[181,182]
**5′ splice site**	Eukaryotes	Linear	U1, U4, U5, U6 snRNP	Marks exon/intron boundary	Familial dysautonomia	[183]
**3′ splice site**	Eukaryotes	Linear	U2 snRNP	Marks intron/exon boundary	Gastrointestinal tumors	[184]
**Polypyrimidine tract (PPT)**	Eukaryotes	Linear	U2AF65	Recruitment of further spliceosomal factors to 3′ splice site	Tetrahydrobiopterin deficiency	[185]
**Branch point sequence (BPS)**	Eukaryotes	Linear	SF1, U2AF65, U2AF35	Marks point of lariat structure in intron as splicing intermediate	Lymphoma, severe pneumonia under SARS-CoV-2	[186,187]
**Splicing enhancer**	Eukaryotes	Linear	SR proteins	Promotes exon inclusion	Myeloma, type-2 diabetes	[188,189]
**Splicing silencer**	Eukaryotes	Linear	SR proteins, hnRNPs	Promotes exon skipping	Myeloma, infant mortality	[188,190]
**miRNA target**	Eukaryotes	Linear	miRNAs	Translational regulation, mRNA decay	Viral infections, cancer	[191,192]
**Smaug recognition element (SRE)**	Eukaryotes, viruses	Linear in stem-loop context	SAM (sterile alpha motif) domain-containing proteins	Regulation of translation, mRNA decay	Myopathy, HBV infection	[41,193–197]
**Localization elements**	Eukaryotes, prokaryotes	(multiple) stem-loops	Staufen, She2p, She3p	Sub-cellular distribution of mRNAs for spatial control of translation	ALS	[49–51,65,82,198]
**AU-rich element (ARE)**	All kingdoms of life	Linear / (diverse)	AUF1 (HNRNPD), HuR (ELAVL1), TIA1, TIAR	Mediate mRNA decay for post-transcriptional control	Cancer, autoimmune diseases, infections	[107,108,199]
**Nucleotide repeat expansions**	Eukaryotes	Transition from linear to stem-loops	TDP-43, PKR, hnRNPs, SR proteins, DROSHA	Localization (in some cases)	ALS, FTD	[57]
**Internal ribosomal entry site (IRES)**	Viruses, eukaryotes	Multiple stem-loops	Ribosome, IRES-*trans* acting factors (ITAFs)	Cap-independent translation	Viral infections, cancer	[63,64]
**Alternative decay element (ADE)**	Eukaryotes	Stem-loop (hexaloop)	Roquin, Regnase	Mediates mRNA decay	Autoimmune diseases, cancer	[38]
**Constitutive decay element (CDE)**	Eukaryotes	Stem-loop (triloop)	Roquin, Regnase	Mediates mRNA decay	Autoimmune diseases, cancer	[200]
**Iron-responsive element (IRE)**	Eukaryotes	Stem-loop	Iron response protein	Modulates translation of genes involved in iron metabolism	Hyperferritinemia cataract syndrome	[23,52]
**RNA thermometers and thermosensors**	Eukaryotes, bacteria	Stem-loop	Ribosome, eEIF1A	Temperature-dependent control of gene expression	Bacterial infections	[45–48,105,121]
**Packaging signal**	Viruses	Stem-loop, three-way junction	Envelope proteins, Capsid protein (Cp), Nucleocapsid protein	Mediates packaging of viral genome through RNA-protein interactions	Viral infections, e.g., Influenza, HIV	[54,55,69]
**G-quadruplexes**	All kingdoms of life	Layers of G-quartets	TDP-43, FMRP	Translational regulation	Infections, neurodegenerative disorders, cancer	[65,144,201,202]
**Sarbecoviral pan-end activating RNA (SPEAR)**	Viruses	Stem-loop	EPRS1	Enhances ribosomal frameshifting and viral translation	SARS-CoV-2 infection	[203]
**Conserved RNA replication element (CRE)**	Viruses	Stem-loop (14-nt loop)	3D^pol^ unit of replicase complex	Mediates replication of viral genome	Infections with enteroviruses and rhinoviruses	[204]
**Exoribonuclease-** **resistant RNA (xrRNA)**	Viruses	Pseudoknot-ring- structure	None	Protects viral RNA from ribonuclease cleavage	Various viral infections	[17,58,205]
**Frameshifting element (FSE)**	All kingdoms of life	Stem-loop	Ribosome	Production of multiple proteins from single RNA sequence	Various viral infections	[97,141,142,206,207]
**Pseudoknot (PK)**	Eukaryotes, viruses	FSE interacting with downstream sequence	Interacting RNA element in *cis*	Translational regulation, protection of RNA	Various viral infections; translational mis-regulation	[97,141,206]
**Kissing stem-loops**	All kingdoms of life	Two stem loops, FSE interacting with downstream stem-loop	Interacting RNA in *trans*	Dimerization of RNA, intramolecular RNA-RNA interactions	Various viral infections	[76,77,208]
**Cyclization element**	Viruses	Complex arrangement of multiple stem-loops, long-range RNA-RNA interactions	Distant RNA region (intramolecular)	Cyclization of genome for replication	Various viral infections	[72–74]
**INF-γ-activated inhibitor of translation element (GAIT)**	Mammals	Stem-loop	Members of GAIT complex	Translational silencing	Potentially dissolves inflammatory responses	[42]

This listing is not complete.

### Linear *cis* elements

Linear *cis* elements were the first regulatory RNAs described [14]. The availability of whole genome sequences means that *cis* elements in potential targets regulated by the respective RNA-binding protein or microRNA in *trans* can be identified. However, degeneration and context-dependency of RNA elements can still obscure linear motifs from detection. Linear RNA elements provide a sequence-specific engagement with *trans*-factors (Table 1 and Figure 1A), that is, proteins, miRNAs, or lncRNAs. With a size of 21-23 nucleotides (nt), miRNAs bind their target sites specifically, whereas classical RNA-binding proteins usually bind 3–10 nt with nM to µM affinity [31]. To provide sufficient specificity and increased affinity, RNA-binding proteins exploit their modular architecture, through which multiple RBD interact with a cluster of linear RNA *cis* elements [12].

Proteins and RNAs exploit linear *cis* elements for target sequence readout. AU-rich elements (ARE) were among the first discovered *cis* elements, and mediate mRNA decay [15]. The 7-nt seed sequence of miRNAs ensures specific target recognition of Ago proteins within the RNA-induced silencing complex. However, flanking regions can contribute to scanning of the RNP on RNA substrates [32]. Start codons of mRNAs interact with protein and RNA components – the ribosome and tRNAs, respectively. The pre-translation initiation complex together with eukaryotic initiation factor 4F (eIF4F) scans the mRNA for start codons, and helicase activity eventually unfolds existing secondary structures [33,34] to facilitate a sequence readout by the contained tRNA. In splicing, multiple protein factors are involved in recognizing splicing enhancers or silencers, the 5′ splice site, and the polypyrimidine tract (PPT) to ensure proper inclusion or skipping of exons. These RNA elements are linear motifs that can be trapped in structures, and structural context provides a feasible mode of regulation beyond the sequence alone. Despite the sequence-specific engagement of RNA-binding proteins in such an architecture, the structural embedment of these motifs is relevant for function and hence listed here for completeness [25,35].

### Stem-looped *cis* elements

Stem-loops (SL) account for most RNA structures found to date. Despite a common architecture, SL structures come in different flavors, arising from internal symmetric or asymmetric loops, bulges, and varying stem lengths [36] (Table 1 and Figure 1A,B). The structural stability of SLs [37] and the higher-order structures of branched SLs add to the variety of tertiary structures [25] (Table 1). Two well-described types of SL *cis* elements are the alternative and constitutive decay elements (ADE [38] and CDE [39], respectively), which initiate mRNA decay through the multi-domain protein Roquin. ADE and CDE comprise a hexa- or triloop that mediates sequence-specific interactions with Roquin in an otherwise shape-driven complex formation. Elements such as the CDE can serve as an ARE in a linear conformation and recruit classical ARE-binding proteins like AUF1 [40]. Sequence-specific recognition is also provided by the Smaug recognition element (SRE) in the context of a hairpin structure, which contributes to mRNA decay and translational regulation [41,42]. Stabilization of the stem decreased the affinity of RNP formation, congruent with altered dynamics of the RNA in the protein-bound form [43]. This observation highlights how RNAs exploit their structural plasticity to specifically engage with subsets of *trans* partners.

The structural integrity of SLs is critical for RNP formation. Hence, conformational transitions based on stem stability are suited to sensing temperature changes. Originally discovered in bacteria [44], RNA thermometers in eukaryotes allow translational adaption in response to heat shock and were presumably adapted from prokaryotes in mammals [45,46]. In rice, global unfolding of mRNAs at elevated temperatures lowered transcript levels and reduced translation [47]. SL elements contribute to translational regulation in the life cycle of *Leishmania* [48]. Localization signals (or zip codes) determine cellular mRNA destinations and facilitate spatial control over translation. These SLs can be recognized by double-stranded RNA-binding proteins [49–51]. Other SL elements have been found in iron-responsive elements [23,52] and in viral packaging signals [53–55]. SLs can be created by mutations, driven by slippery transcription, and lead to nucleotide repeat expansions causing neurodegenerative disorders [56,57].

### *Cis* elements with higher-order structures

SLs, bulges, and single-stranded regions are key building blocks for higher-order structures in RNA (Table 1 and Figure 1B). The modular architecture of RNA often requires sequential folding of individual elements to properly engage with each other through tertiary contacts. Pseudoknots, such as the frameshifting element (FSE) in viruses, are SLs interacting with single-stranded regions through loop bases. Exoribonuclease-resistant RNAs (xrRNAs) are viral elements with nuclease resistance based on extraordinary stability of the tertiary structure formed by pseudoknots that assemble in a protective ring around the RNA in 3′ of itself and prevent the 5′ to 3′ progression of ribonucleases [58]. Steckelberg et al. proposed that the resistant structure forms during 5′ degradation, instantly creating the tertiary structure contact sites. Instead of pseudoknots, some viruses contain three-way junctions, which exhibit the same RNA-stabilizing effect [59]. Pseudoknots and three-way junctions prevent degradation in viral RNAs and have been suggested to contribute to maturation of mRNAs [58].

IRES allow cap-independent translation of mRNA, for example, through mimicking tRNA-like structures [60]. Viral IRES compensate the deficit of a virus’ own translation machinery and hijack host ribosomes for viral translation. IRES also occur in eukaryotic mRNAs that encode regulators of apoptosis and growth factors, offering a route for translation under stress conditions when conventional cap-dependent translation might be compromised, such as under hypoxia [61]. IRES-mediated production of the transcriptional regulator p27 was speculated to compete with viral IRES under infection [62]. Kullmann et al. further showed that HuR and HuD proteins negatively regulate IRES activity and prevent ribosomal translation [62]. In contrast, the lncRNA TRMP (TP53-regulated modulator of p27), which can be induced by the tumor-suppressor p53 and promotes cell cycle progression, indirectly regulates p27 IRES activity by competing for binding of pyrimidine tract-binding protein 1 (PTBP1) [63]. Kolupaeva and colleagues showed that PTBP1 recognizes consensus sequences in a structured context and stabilizes the IRES in a ribosome-competent conformation [64]. The p27 IRES is thus a paradigm for an RNA element anchor for a *cis*-*trans* network that integrates multiple signals (Figure 1C) [63].

Localization signals can be more complex than single hairpins. The protein TDP-43 (TAR DNA-binding protein of 43 kDa [65],) binds mRNA G-quadruplexes and targets them to distal neurites. Ishiguro et al. observed a marked loss in affinity to G-quadruplexes for a TDP-43 point mutation frequently found in patients with amyotrophic lateral sclerosis. Functional *cis*-*trans* pairs rely on specific and fine-tuned interactions, and mutations on either side can perturb complex formation. G-quadruplexes are composed of four strands of consecutive guanines in varying topology, with the bases interacting through Hoogsteen bonding in three planes stacking upon each other [66]. In plants, G-quadruplexes enhance mRNA stability in cold adaptation, thus regulating transcript levels instead of translation as observed in the heat shock response [67]. Kharel et al. reported that G-quadruplexes were more commonly induced by stress and were positive, yet reversible, regulators of mRNA stability [68].

In addition to the SL structures discussed above, viral packaging signals can be structurally more elaborated. Keane et al. determined a high-resolution nuclear magnetic resonance (NMR) structure of the 155-nt HIV packaging signal [69]. A tandem three-way junction formed the core element and integrated long-range RNA-RNA interactions to delicately expose key residues for RNP formation. The RNA structure itself encoded all determinants for translation abrogation, genome dimerization, and packaging through protein binding. Hence, this RNA is a regulatory hub connecting processes in the viral life cycle that require orchestrating.

### Discontinuous *cis* elements

Two *cis* elements that are distant in sequence can form a structural unit that is essential for function [70] (Table 1). Such interactions can span hundreds to several thousands of nucleotides [70,71]. *Flaviviridae* and other virus taxa frequently use these intramolecular RNA–RNA long-range interactions for genome replication [72–75]: Highly structured regions at the 5′ and 3′ termini interact through complementary sequences and lead to cyclization of the RNA genome, which is required for heightened activity of the viral RNA-dependent RNA polymerase (RdRP). Such processes require substantial structural rearrangements, but detailed mechanistic insights are currently lacking. Viruses also exploit distant RNA–RNA interactions to modulate translation. For instance, *Pea enation mosaic virus* (PEMV) contains a T-shaped RNA *cis* element at its 5′ end, which engages with a SL structure within the coding sequence through a kissing-loop [76]. This newly formed element binds to the ribosomal subunits and is crucial in boosting viral protein translation. A kissing-loop interaction composed of two hairpins from the 5′ and 3′ UTRs of *Barley yellow dwarf virus* (BYDV) was suggested to regulate access of ribosomes to the translation initiation site [77]. The interaction therefore controls the translation rate through constant formation and disruption of the structure. Distant but functionally coupled *cis* elements are most frequently found in viruses.

### Regulatory units composed of multiple *cis* elements

RNAs often exert their functions not through one *cis* element, but via multiple copies of the same element or structurally redundant versions thereof (Table 1 and Figure 1C). These elements can be structurally independent and have an additive functional effect [78–80]. Evolutionarily, this constellation renders these regulatory clusters more robust to perturbations induced by mutations and allows fine-tuning of the concerted output [80,81]. In budding yeast, localization of the *ASH1* mRNA to the bud tip is mediated by four independent localization elements, each of which are sufficient for proper localization [82].

*Cis* elements may also work in a cooperative manner, as demonstrated for multiple miRNA response elements [83]. In addition, cooperativity has been suggested for structured RNA elements. For example, a tandem SL showed cooperative behavior with a-in sequence-distant SL for binding to the Roquin and Nufip proteins [84]. Furthermore, the binding of multiple Roquin molecules to six distant SLs in the *Nfκbid* 3′UTR cooperatively regulated mRNA levels [81]. Although the mechanism is unknown, these observations imply that structural rearrangements of the RNA bring all SLs in proximity. In *Caenorhabditis elegans*, 3′UTR-mediated down-regulation of the *die-1* transcription factor is required for formation of the left/right axis within gustatory neurons. Three 3′UTR regulatory elements showed a partially additive and redundant effect on regulation efficiency [85]. However, full regulation could not be achieved by three copies of the same regulatory element, highlighting the complex interplay within *cis* element cassettes.

The occurrence of multiple *cis* elements is frequently observed, but mechanistic insights into their precise (structural) interplay are limited. Cell-based experiments often experience ambiguous or gradual effects, hampering data interpretation [86]. In addition, *in vitro* analysis of *cis* element interplay revealed how the combined binding of two RNA elements by multi-domain proteins integrates sequence and shape recognition to increase specificity in the target search [87,88]. Furthermore, additional *cis* elements might not be directly involved in *trans* factor-binding but are required for the integrity of an entire regulatory hub to ensure proper structural arrangement of the binding site [89] (Figure 1D).

## Transient structures in RNA elements

**Figure 2:**
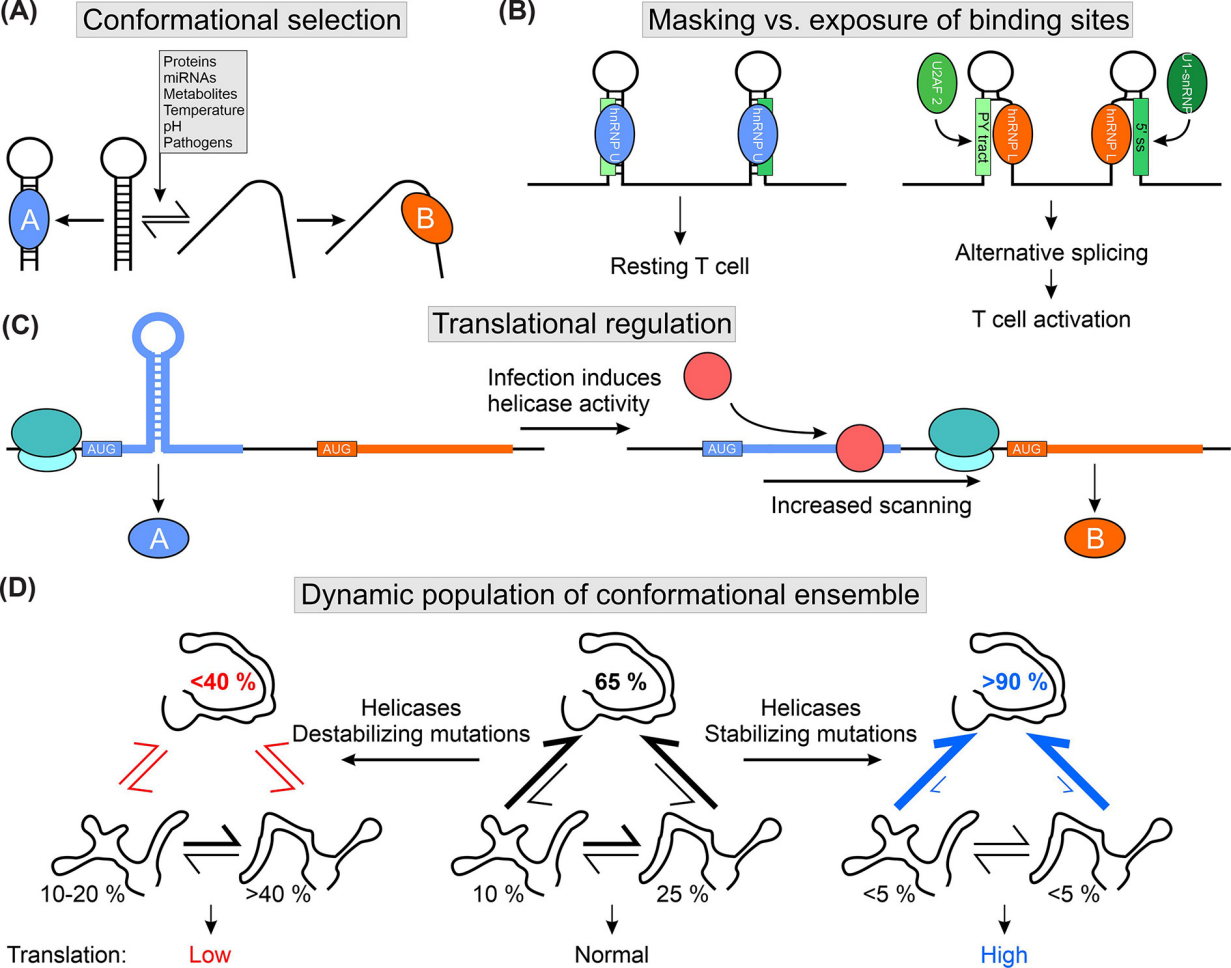
The effect of transient RNA structures on *cis-trans* pair formation (**A**) Schematic of conformational equilibria of an RNA stem-loop (adapted from [25]). Structural changes can be induced by *trans* factors, environmental stimuli, or by infections. Conformation-specific exposure of *trans*-factor binding sites leads to distinct formation of *cis*-*trans* complexes. Often, this equilibrium is influenced by the abundance of *trans* factors, e.g., proteins A and B. (**B**) The competition of hnRNP U and hnRNP L regulates alternative splicing in *MALT1* pre-mRNA (adapted from [25]). hnRNP U binds and stabilizes two stem-loop structures, whereas hnRNP L partially unwinds the RNA, exposing two binding sites-the poly-pyrimidine tract (PY tract) and the 5′ splice site (5′ ss)-which are recognized by U2AF2 and U1 snRNP. Binding of both proteins causes exon inclusion and alternate protein production, leading to T cell activation. (**C**) RNA structure guides start codon selection and modulates translation in *Arabidopsis thaliana* (adapted from [101]). Hairpin structures slow ribosomal scanning and lead to the translation of protein A (blue ORF). Upon infection, helicases (red) resolve RNA structures, leading to increased scanning through the ribosome and translation of the downstream ORF (orange), i.e., protein B. Downstream ORFs encode immune-relevant proteins. (**D**) Translation regulation of *Csde1* through a dynamic equilibrium of 5′UTR RNA structures (adapted from [6]). *In vivo* the major conformation (65%) controls translation efficiency. Helicases and point mutations can modulate the conformational ensemble through stabilizing or destabilizing single conformations, thereby shifting the equilibrium and tuning translation efficiency (blue and red equilibria).

Distinct RNA folds (for example, tetraloops [90,91]) can adopt rigid structures. However, most RNAs sample multiple conformations in a dynamic, but not random-equilibrium [92]. These changes can be intrinsic, provided several structures are stabilized by a similar free enthalpy. Often, structural changes are induced, for example, through changes in temperature, pH, or ligand binding (Figure 2A). Mutations found as drivers of diseases can lead to alterations in RNA folding by altering (distant) base-pairing patterns [22], and insights into structural and stability changes of RNAs through modifications like m6A have been described recently [93–95]. Proteins can cause major structural rearrangements upon binding and might therefore modulate interactions with further *trans* factors.

Dynamic transitions between multiple conformations allow the RNA to respond to and integrate different stimuli. Ultra-conserved 5′UTR elements provide multiple RNA structures for cell type-specific and fine regulation of gene expression [6] (Figure 2D). Similarly, mRNA structures were found to change during development [1]. Consequently, the interaction network varies depending on the dominant RNA conformation. The 5′ hairpin of 7SK RNA was experimentally shown to exist in four distinct conformations, of which only one stable state could interact with the protein HEXIM [96]. Moreover, altering the equilibrium of the four RNA states through mutations disturbed complex formation in molecular dynamics simulations. For the FSE of SARS-CoV-2 (the virus causing coronavirus disease 2019 (COVID-19)), multiple conformations showed varying effectiveness in frameshifting through altered interactions with the ribosome [97]. RNA can thus select its *trans*-binding partners through structure. Contrary to this, proteins can modulate and shift the conformational equilibrium; for instance, AUF1 and Roquin compete for the same RNA element in the *UCP* 3′UTR which shuttles between a linear ARE form and a stable CDE hairpin [40] (Figure 2A). During splicing, hnRNP U and hnRNP L bind the same SL element in the *MALT1* mRNA albeit with opposing effects: hnRNP U leads to increased stability of the structured RNA, whereas hnRNP L unfolds the element and exposes a second *trans* factor binding site, leading to exon inclusion and alternative splicing through engagement of U2AF 1 and U1 snRNP [25] (Figure 2B). For both *UCP* and *MALT1* RNA two proteins read out the conformational state of a single RNA element and modulate it. During translation, RNA structures are diminished by the ribosome [1]. Regulation of *trans* factor binding sites is a key feature of RNA structure and is the rate-limiting step in miRNA-mediated mRNA cleavage [98].

Deprivation of RNA structure is exploited by respiratory viruses as a strategy for evading the human immune system: Rigby et al. observed a decrease in RNA secondary structure content in influenza A virus and SARS-CoV-2 over time and a correlation with large pandemics [99]. The affected transient RNA structures (template or t-loops) form during viral RNA synthesis and interact with the viral RNA polymerase. T-loops likely modulate viral RNA production and activate the immune response by RIG-I through aberrant ribosome stalling [100]. Dynamic shuttling between structure formation and its absence can further regulate translation [77]. Translational reprogramming in *Arabidopsis thaliana* relies on the availability of hairpin structures 3′ of upstream start codons [101]; infection-induced helicases resolve these SLs and facilitate ribosomal access to downstream open reading frames, which typically encode immune-relevant proteins (Figure 2C).

RNA structure allows highly specific RNP formation. As such, viruses frequently mimic the structure of tRNAs to hijack ribosomes, for example, in IRES or the HIV-1 5′UTR [102]. Therefore, tRNA-like structures exploit multiple conformations [103]. Changes in RNA structure frequently serve as a molecular switch, such as in translation regulation in bacteria [104] or during infection with *Vibrio cholera* when a temperature increase in the human host exposes the Shine Dalgarno sequence within an RNA thermometer structure [105]. In the latter example, the RNA unfolds at 37 °C and initiates translation of the transcriptional activator ToxT, leading to the production of virulence factors like cholera toxin. Transient RNA structures are also used by viruses throughout their life cycle to regulate replication, transcription, and translation [106] and by mammals for heat adaptation [89].

## Perturbations in RNA structure and associations with diseases

RNAs have been linked with numerous diseases including cancer, autoimmune and neurodegenerative diseases, and infections (Table 1) [54,57,107,108]. Changes in RNA sequence, modification [109], or structure cause a gain-of-function or loss-of-function phenotype. Although sequence changes often translate into mutated and nonfunctional proteins, alterations at the modification or structural level can have multiple effects.

Neurological disorders can be caused by nucleotide repeat insertions, such as in fragile X-associated tremor/ataxia syndrome and Huntington’s disease, reviewed in [110]. These repetitive elements form hairpin structures and create new targets for proteins not previously associated with the respective RNA. Recruitment of muscleblind proteins [111], which are splicing regulators, to CCUG repeats has been associated with myotonic dystrophy type 1 and 2 [112]. These insertions arise from Alu elements and diverged in the evolution of monkeys and apes [113]. Alu elements inserted together with LINE-1 in the ATXN10 gene created AUUCU repeats, which is a recognized cause of spinocerebellar ataxia type 10 [56]. Retrotransposons hence evolved as a source of various pathogenic repeat insertions. Consequently, experiments were designed to bind and block these repetitive RNA structures [112]. Cellular stress is another cause of neurodegenerative disorders, and ferroptosis induced by altered m6A levels contributes to brain damage [94].

Guanine-rich repetitions can form G-quadruplexes or hairpins with internal loops, with GGGGCC hexanucleotide repeat expansions sequestering proteins from the cellular pool (for example, TDP-43 [65]) or leading to R-loops, which subsequently cause amyotrophic lateral sclerosis [57]. In cancer, conformational heterogeneity of G-quadruplexes modulates the levels of tumor suppressor protein p53 or causes translational repression [114,115]. Recently, the splicing regulating SR-protein SRSF1 was shown to dissolve G-quadruplexes [116], and this finding linking splicing and aberrant G-quadruplex formation could facilitate new therapeutic strategies. In addition, prometastatic splicing can be promoted by SNRPA1, which interacts with newly discovered splicing-enhancing structured RNAs in the proximity of exons [117]. Elevated exon inclusion is connected to increased breast cancer prevalence and can be partially rescued by classical splicing regulators, that is, morpholinos. Morpholinos are nucleic acid analogs that bind to their RNA targets in a sequence-specific manner and are frequently used as a tool in cell biology for studying splicing and gene regulation [118].

Not only the structure of an RNA can be relevant for *trans* factor binding, but also its ability to resolve. The structural lability of a SL in exon 10 of *tau* governs splicing [119]. Mutations associated with dementia and Parkinson’s disease lead to reduced stability of the RNA fold [120] and cause increased exon 10 inclusion [37]. David et al. observed that in *Leishmania* infections melting of a 3′UTR-located SL PPT favors translation at elevated (human) body temperatures [48], thereby promoting infection. RNA thermometers regulate the expression of virulence factors and toxins [105,121], and are thus perfect examples for the loss of structure having a functional impact.

Pathogens frequently use programmed RNA structural transitions to progress in their infectious life cycle. However, conformational changes induced by spontaneous mutations can perturb homeostasis and cause disease [23]. For example, SNPs in a 5′UTR hairpin structure disrupt the fold and render the RNA invisible to iron-responsive proteins. Lack of protein binding is associated with hyperferritinemia cataract syndrome [23]. The UTRs of mRNAs are especially sensitive to point mutations because they are rich in mRNA regulatory elements often located in secondary structure. One option for controlling mRNA levels and decay is miRNA binding to the 3′UTR. SNPs found in 3′UTR structures alter miRNA binding and contribute to diseases [21]. Tools are available for predicting and identifying SNPs that cause structural variations-also called RiboSNitches-and for differentiating these SNPs from silent mutations [122].

**Figure 3:**
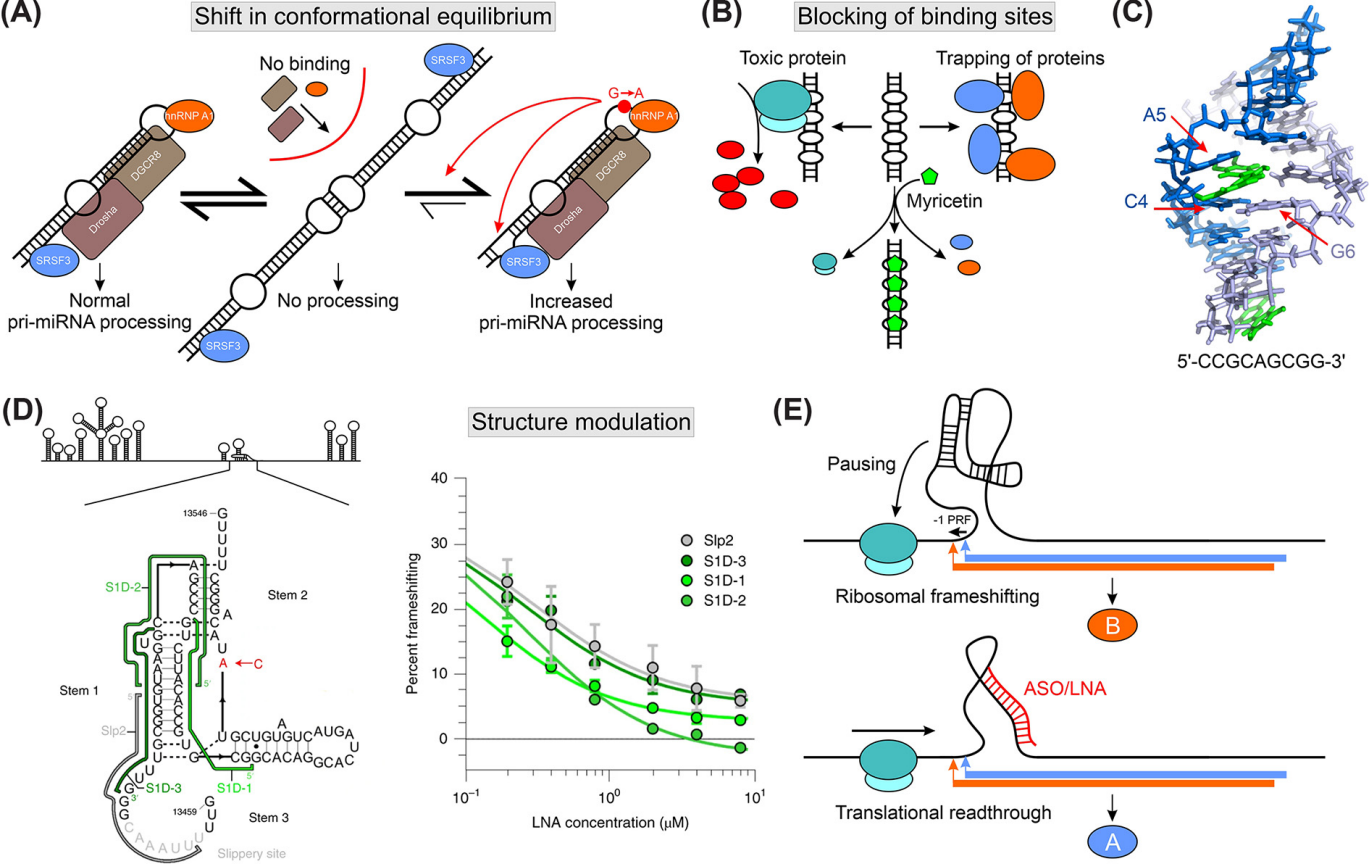
RNA structure as a driver of diseases and drug strategies (**A**) pri-miRNA-30c binds SRSF3 (blue), hnRNP A1 (orange), and Drosha and DGCR8 for processing (adapted from [22]). The apical loop mediates dimerization, which obscures the protein-binding sites and prevents RNA processing. A cancer-associated G-to-A point mutation in the loop shifts the equilibrium to the monomeric RNA form (red arrow). Furthermore, structural rearrangements in the lower stem facilitate SRSF3 binding (red arrow). Enhanced protein binding leads to an increase in pri-miRNA processing. (**B**) CAG repeat expansions form repetitive stem-bulge structures, sequester and trap cellular proteins (blue and orange), and cause translation of prionic (toxic) polypeptides (red). The flavonoid myricetin (green) masks stem-bulge structures from protein binding, thereby preventing its toxic effects (adapted from [136]). (**C**) Structure of a CAG-repeat RNA element in complex with myricetin (adapted from [136]; PDB: 5XI1). Myricetin (green) intercalates in the RNA duplex and pushes A5 from strand 1 (blue) out of the helix. The flavonoid interacts through π-π stacking with the C4-G6 base pair, thereby connecting both RNA strands (blue and light blue). (**D**) RNA secondary structure scheme of the SARS-CoV-2 genome and frameshifting element (FSE) highlighted in zoom-in. The experimentally determined secondary structure is shown together with the ribosome slippery site and interaction sites of tested ASOs (adapted from [141]). Frameshifting efficiency plotted against LNA concentration from *in vitro* frameshifting assay (taken from [141] under license 5794170311982). The colors of tested LNAs correspond to binding sites indicated in the secondary structure on the left. A significant reduction in frameshifting is observed at nM-concentrations. (**E**) Model for mode of action of ASO targeting the FSE (adapted from [141]). The FSE structure causes pausing of the translating ribosome, allowing a frameshift within the slippery site (production of protein B, orange). Binding of ASOs alters the FSE structure and causes a translational read-through to produce protein A (blue).

SNPs in miRNAs can have detrimental effects on gene regulation. A G-to-A loop mutation in the pri-miRNA-30c shifts the equilibrium from a dimeric form to the monomer through partial destabilization of the RNA stem (Figure 3A) [22,123]. Consequently, the loop exposes a hnRNP A1 binding site, which causes increased miRNA processing. Originating from an oncogenic miRNA cluster, this mutation is frequently observed in cancer. Similarly, misregulation of the miR-17-92a cluster (oncomir-1) was found to be carcinogenic [124]. The cluster contains six miRNAs that exhibit differential processing efficiency throughout development. Extensive secondary structures prevent efficient processing by the Drosha-DGCR8 complex, and the miRNAs only mature upon rearranging structure. Conformational changes are likely induced by *trans* factors or an intrinsic pH-sensitive RNA element [124,125].

Sequence mutations in avian influenza viruses also mediate RNA structural changes. The viruses exploit these conformational changes in switching from low to highly pathogenic forms [126]. Viruses have developed further sophisticated RNA elements to promote infection [58] and packaging [75] and readers interested in this topic are referred to the more specialized literature [127,128].

## RNA structure as a drug target

Over the past decade, efforts have been made to target (dynamic) RNA structures using small molecules [129,130]. In 2021, the first RNA-targeting drug, Risdiplam, was approved [131] for treating spinal muscular atrophy. Risdiplam is a splicing modifier that leads to exon inclusion in the SMN (survival of motor neuron) mRNA and thereby increases the amount of functional SMN protein in patients. The recent COVID-19 pandemic brought RNA structures as drug targets into public focus. The most promising attempts to target RNA structures address viral infections, with numerous studies evaluating the applicability of small molecules [132], locked nucleic acids (LNA) [133], or antisense oligos (ASO) in combating viral infections (Figure 3B). An additional covalent bridge within the sugar moiety confers nuclease-resistance to the LNA, which is essential for drug applications. ASOs bind their targets in a sequence-specific manner through perfect complementarity and can block downstream functions or initiate RNA decay [134]. Phosphonate-peptide nucleic acids offer an alternative to ASOs, providing greater stability and more effective delivery in cells [135].

An appropriate strategy for preventing or curing diseases can be blocking of the binding sites of RNA-binding proteins, thereby hindering *trans* factors from accessing the RNA (Figure 2B). Neurological disorders exhibit their pathogenic phenotype through excessive (and unspecific) recruitment of proteins to the nucleotide repeat expansions-trapping the proteins in macromolecular agglomerates-or by translation of aberrant and toxic polypeptides (Figure 3B) [136]. Transcript levels do not necessarily correlate with (toxic) gene product levels. Hence, for the treatment of neurodegenerative diseases, targeting RNA structures is a pivotal option for avoiding aberrant protein production from the outset [137,138]. The flavonoid myricetin binds repetitive elements of stem-bulges and prevents translation through the ribosome and sequestration of proteins (Figure 3B and C). Myricetin binding has demonstrated positive effects in cell-based assays [136]. A similar effect was observed by Oldani et al. for G-quadruplex-mediated aggregation of TDP-43 [139].

The high mutation rates of viruses and associated drug resistance mean highly conserved RNA secondary structures are the targets of choice in developing therapeutics for these micro-organisms [53,138]. The FSE is a reasonable drug target in RNA viruses because the FSE leads to pausing of the scanning ribosome and causes a −1 frameshift, thereby enabling translation of a second open reading frame. Consequently, a functional impairment in the FSE can down-regulate viral protein translation. Aminoquinazoline derivatives bound the SARS-CoV-2 FSE at 60 µM and despite the moderate affinity showed an inhibitory effect in reporter assays [140]. Zhang and co-workers found that ASOs modified with LNAs effectively reduced viral replication in cells at concentrations of 100 nM by binding to stem 1 of the FSE (Figure 3D) [141]. ASOs unwind the FSE structure, facilitate ribosomal readthrough, and favor translation of an alternate protein (Figure 3E). LNAs targeting the HIV-1 FSE also proved to be effective modulators of the FSE structure, and alternated frameshifting efficiency *in vitro* [142]. Other promising viral targets are IRES and packaging signals [16]. LNAs successfully bound the packaging signals of Influenza virus and SARS-CoV-2 and prevented infection and transmission of viral particles in rodents [53].

In addition to modulating RNA structure and blocking *trans* factor binding sites [136], stabilizing the structure of RNA can prevent adoption of a preferable (and functional) conformation. For example, in HIV-1 packaging, a labile RNA helix needs to unwind for binding by the nucleocapsid protein, and this mechanism can be perturbed by small molecules [54,143]. Similarly, stabilization of G-quadruplex structures attenuated SARS-CoV-2 infections [144].

Although the strong plasticity and heterogeneity of RNA poses a challenge in structure determination, heterogeneity offers access to drug design as usually only a limited number of conformations are functionally active. Therefore, an elegant but challenging approach is to target single RNA conformations specifically, for example, by shifting the equilibrium towards low-populated inactive states. Conformational equilibria can readily be analyzed by solution-based methods, such as NMR spectroscopy, *in vitro* [145]. Murchie et al. improved a series of inhibitors that bind to the HIV-TAR RNA, which is required for induction of viral translation after complex formation with the Tat protein [146]: *In vitro*, the inhibitors trapped the RNA in a conformation incompetent for Tat binding at a K_i_ > 100 nM. For the IRES of Hepatitis C virus, modulating the positions of two RNA helices relative to each other effectively aborted IREs-mediated translation through undocking of the ribosome [16].

## Methods for capturing RNA *cis* element structures and dynamics

Reasonable progress has been made in predicting new structured RNA elements. However, structure prediction of RNA is still demanding, and computational methods remain the method of choice for identifying *cis* elements, either as a stand-alone approach or combined with genomics data [147,148]. Morandi et al. developed the SHAPEwarp pipeline which identifies structurally related or similar regions in RNAs based on SHAPE data [149]. Ignoring the primary sequence, SHAPEwarp allows an unbiased search and enables identification of conserved RNA folds, which facilitates classification and identification of *cis* elements. However, structural information of RNA elements is still a prerequisite for the discovery of new *cis* elements. The dynamic nature of RNA structures imposes challenges on structure determination and subsequently the three-dimensional atom-resolved depiction of *cis* elements. Numerous *in vitro* methods have been developed to reveal RNA folding, mostly addressing secondary structure (Table 2). Gel-based methods-for example, in-line probing (ILP) or hydroxyl-radical probing (OH probing)-report on secondary structure or solvent accessibility [78,150,151], and have been used to track conformational changes of RNA *cis* elements in the presence of ligands or proteins, or their binding sites, collectively termed footprinting [152]. These so-called probing assays can provide information on binding affinities and give mechanistic insights [153]. For instance, an increased affinity of the methyltransferase ribozyme MTR1 for O6-methylguanine compared with guanine was observed by ILP. In all probing assays, chemicals or enzymes cleave the RNA, yielding a characteristic pattern on a gel. A broad selection of protocols and probes is available [154], but the methods come with size and resolution limitations of the probed RNAs.

**Table 2:** Methods to determine RNA secondary and tertiary structures *in vitro* and *in vivo*. n.a., not applicable

Method	Readout	Structural information obtained	*In vitro* specifications	*In vivo* specifications
**DMS probing / Chemical probing**	Reactivity profile	Single-stranded / unstructured regions	Analysis by gel or sequencing Detection of conformational changes after ligand binding Base-specific selection of chemical agents [209,210]	DMS-MapSeq Sequencing-based For high- and low-abundance RNAs [210–212]
**In-line probing**	Cleavage	Single-stranded / unstructured regions	Analysis by gel or sequencing Detection of conformational average [78,150]	n.a.
**SHAPE**	Reactivity profile	Single-stranded / unstructured regions	Analysis by gel or sequencing Detection of conformational average Well suited for large RNAs [97]	icSHAPE (in-cell SHAPE) For high- and low-abundance RNAs Compatible with several probing reagents and cell types [172,213]
**Hydroxyl radical probing**	Cleavage	Solvent-accessible regions	Analysis by gel or sequencing Detection of conformational average [214,215]	n.a.
**RNase cleavage assay**	Cleavage	Depending on RNase used single-stranded or duplex regions; solvent-exposed regions	RNase T1, T2, A, V1, S1/P1, J1, H Analysis by gel or sequencing Detection of conformational average [151,216,217]	n.a. (can be mimicked with cell extracts)
**Mutate-and-map**	Reactivity profile	Single-stranded / unstructured regions, reconstruction of contacts through comparative high-throughput mutations, changes in tertiary contacts	Sequencing-based Tertiary model building possible with computational tools [163]	icM^2^ (in-cell mutate-and-map) Transfection of mutated plasmid into cells Can be mimicked with cell extracts [6,218]
**RING-MaP**	Reactivity profile	Tertiary contacts, discovery of low-populated conformations Also available for secondary structure determination [219]	Sequencing-based Computational clustering of co-occurring mutational events [157]	n.a.
**Multiplexed OH Cleavage Analysis with paired-end sequencing (MOHCA-seq)**	Reverse transcription stop	Secondary structure, tertiary contacts	Powerful for tertiary structure modelling, e.g., with FARFAR [162,164]	n.a.
**Cross-linking ligation and sequencing of hybrids (CLASH)**	Sequence of ligated RNA fragments based on interactions	Interacting RNA partners, interaction sites	n.a.	Well-suited for miRNAs, piRNAs Sequencing-based Protein-assisted [220,221]
**Sequencing of psoralen cross-linked, ligated, and selected hybrids (SPLASH)**	Sequence of ligated RNA fragments based on interactions	Interacting RNA partners, interaction sites	n.a.	Applicable to all types of RNAs Sequencing-based [222]
**Psoralen analysis of RNA interactions and structures (PARIS)**	Sequence of ligated RNA fragments based on interactions	Base pairs, tertiary interactions	n.a.	Sequencing-based Structural information of entire transcriptome possible [223]
**Mapping RNA interactome *in vivo* (MARIO)**	Sequence of ligated RNA fragments based on interactions	Base pairs, mainly tertiary interactions	n.a.	Protein-assisted Sequencing-based [224]
**Cross-linking of matched RNAs and deep sequencing (COMRADES)**	Sequence of ligated RNA fragments based on interactions	Base pairs, tertiary interactions	n.a.	Sequencing-based Enrichment of selected RNAs Detection of multiple conformations [225]
**Spatial 2′-hydroxyl acylation reversible crosslinking (SHARC)**	Sequence of ligated RNA fragments based on interactions	Tertiary interactions	n.a.	Sequencing-based Use of crosslinkers as molecular rulers Detection of multiple conformations [226]
**AFM / optical tweezers**	Change in contour length under force	Overall secondary structure (single-stranded and duplex regions); folding pathways	Single-molecule technique Requires low amounts of material [22,167,205]	n.a.
**SAXS**	Scattering profile	Geometric parameters (D_max_, R_g_)	Low resolution Captures full conformational space as average Flexible with respect to size [78,166,227]	n.a.
**Cryo-electron microscopy (cryo-EM)**	Contrast image	Low-to-high-resolution structures	Optimization of construct Does not always capture full conformational space [141,160,228,229]	n.a.
**Cryo-electron tomography (cryo-ET)**	Contrast image	Low / medium resolution of macroscopic structures	n.a.	Suitable for large RNAs Low resolution No solution method [230,231]
**Nuclear magnetic resonance (NMR)**	Imino protons Structures	Double-stranded / structured regions High-resolution, atom-resolved structures	Size limited Possible detection of multiple (low-populated) states [40,69,78,161]	Requires introduction of large amounts of exogenous RNA into cells Read out of imino protons is straightforward [232–234]
**Crystallography**	Diffraction	High-resolution, atom-resolved structures	Optimization of construct and crystallization conditions Does not always capture full conformational space [235]	n.a. (only available for proteins) [236]

Sequencing-based approaches overcome the limitations of probing-based assays [154] and datasets can be bioinformatically deconvoluted to detect low-populated conformational states of RNA elements [155–157], which can otherwise only be revealed by laborious high-resolution structural biology methods such as NMR spectroscopy or cryogenic electron microscopy (cryo-EM) [103,158–160] supplemented with computational approaches [161]. Sophisticated protocols even allow tertiary structure information to be deduced [157,162,163], as evidenced with the RING-MaP technique. These methods are especially powerful in combination with a bioinformatic data processing and analysis [164]. Besides experimental approaches, pure computational methods can be used to predict RNA structures and changes upon mutation [165].

The large structural plasticity of RNAs can be captured by methods like small-angle X-ray scattering (SAXS) or atomic force microscopy (AFM) [166,167]. Optical tweezers can be exploited to unravel folding pathways and intermediates [22] and have been used for studying the dynamics of viral structural RNA elements [168]. Optical tweezers are unique as they apply mechanical force to the RNA. This feature was used by Neupane et al. in revealing that the SARS-CoV-2 pseudoknotted FSE exists in two distinct conformations and additional alternative structures, characterized by individual folding pathways and stabilities [168]. The conformational propensities described therein have implications for ribosome binding, i.e., frameshifting and drug design.

RNA structures *in vivo* often differ significantly from those determined *in vitro* [169]. In addition, the overall degree of structure *in vivo* can be higher than expected, for example, in 3′UTRs [1]. However, *in vivo* structure determination is even more challenging than *in vitro* determination, owing to the low abundance of target RNAs, their heterogeneous decoration with proteins, and assay bias [170]. Varying interactions with *trans* factors throughout an RNA life cycle can significantly alter RNA structure. To obtain excess to these dynamic RNA interactions and conformations, *in vitro* reconstitution of RNPs is often a feasible approach. Consequently, careful planning of experiments and data interpretation is required, e.g., to mimic near *in vivo* conditions of RNPs as done for the ribosome which comprises up to 50 different proteins [171]. Nevertheless, established *in vitro* methods have advanced into frequently used *in vivo* probing approaches, including in-cell SHAPE and in-cell mutate-and-map [6,172] (Table 2), allowing detection of single isoforms. Elaborated computational tools evaluate differential probing results from varying cellular conditions [173]. Thereby, the changing availability of RNA structure can be assessed, and structurally flexible regions are revealed. However, although the methods toolbox for *in vivo* RNP detection is constantly increasing, the underlying *cis-trans* code often remains elusive or requires extensive (*in vitro*) downstream work. A fundamental problem is the unpredictability of spatiotemporally dependent and resolvable RNA structures in cells.

## Conclusion and outlook

The discovery of currently unknown *cis* elements as regulators in gene regulation is a critical future task in molecular biology, especially when linked to diseases. This also includes the identification of regulatory motifs in dynamic RNA regions exhibiting chemical exchange and therefore potentially masking such elements from their detection. The development of sophisticated structure probing techniques means we can now determine secondary (and to some extent, tertiary) structures of medium-to-large RNAs *in vivo* [174,175]. In some cases, individual conformational states have been described, and similar success is expected in other cases of transiently folded or multistate *cis* elements. However, this approach requires computational deconvolution of data, which is not trivial to implement on a routine basis [156]. We expect that ongoing progress will increase the sensitivity of wet lab protocols so that low-populated and short-lived RNA species can frequently be detected. Improved computational processing pipelines will allow structural characterization of heterogeneous RNA populations from such datasets. In turn, these advances will pave the way toward 4D RNA structural biology, where structural changes of RNA throughout its life cycle will be monitored, induced by alterations of the cellular environment or external stimuli [52,176,177]. In light of this, quantitative methods for describing RNA structure populations will reveal the impact of disease-relevant mutations or epitranscriptomics [109,178] on RNA structural equilibria in cells in general, but especially on the level of *cis*-regulatory elements. Consequently, downstream effects on the RNA interactome will be monitored and related to functional consequences, mostly mediated by relevant RNA-binding proteins, many of which may still have to be determined for the various types of folded RNA elements. Similarly, the contribution of intrinsically disordered regions (IDRs) or unstructured linkers within proteins is now being realized with respect to their potency in regulating the affinity to target RNA sites. Fine-tuning of protein concentrations, their posttranslational modifications, and post-transcriptional RNA modifications likely steer RNP composition and fate, but knowledge in this area is still limited.

The full potential of RNAs, particularly the regulatory motifs, as drug targets is yet to be explored. However, considerable progress has been made, and RNA structures have been successfully targeted by small molecules that alter RNA structure, thereby preventing RNP formation [137,138]. On a broader basis, miRNAs have been exploited as potential drug candidates to mediate mRNA decay or block protein-binding sites [179]. However, the lower chemical complexity of RNA compared with proteins and its inherent structural dynamics mean addressing RNA structures specifically remains challenging. Therefore, reducing off-target effects by fine-tuning the composition and concentration of RNA-targeted drugs will be essential. Besides its pharmacological relevance, modulation of RNA structures will evolve as a bio-engineering tool, as demonstrated for translational regulation [180].

## Abbreviations

ADE: alternative decay elements
AFM: atomic force microscopy
ARE: AU-rich elements
BYDV: Barley yellow dwarf virus
CDE: constitutive decay elements
cryo-EM: cryogenic electron microscopy
FSE: frameshifting element
IDR: intrinsically disordered regions
ILP: in-line probing
IRES: internal ribosome entry sites
LNA: locked nucleic acids
n.a.: not applicable
NMR: nuclear magnetic resonance
PEMV: Pea enation mosaic virus
PPT: polypyrimidine tract
RBD: RNA-binding domains
RdRP: RNA-dependent RNA polymerase
SAXS: small-angle X-ray scattering
SL: stem-loops
SNP: single nucleotide polymorphisms
SRE: Smaug recognition element
UTRs: untranslated regions
xrRNAs: exoribonuclease-resistant RNAs
